# Advances in cellular resolution microscopy for brain imaging in rats

**DOI:** 10.1117/1.NPh.10.4.044304

**Published:** 2023-11-30

**Authors:** Su Jin Kim, Rifqi O. Affan, Hadas Frostig, Benjamin B. Scott, Andrew S. Alexander

**Affiliations:** a Johns Hopkins University, Department of Psychological and Brain Sciences, Baltimore, Maryland, United States; b Boston University, Center for Systems Neuroscience, Department of Psychological and Brain Sciences, Boston, Massachusetts, United States; c Boston University, Graduate Program in Neuroscience, Boston, Massachusetts, United States; d Boston University, Neurophotonics Center and Photonics Center, Boston, Massachusetts, United States; e University of California Santa Barbara, Department of Psychological and Brain Sciences, Santa Barbara, California, United States

**Keywords:** calcium, head-mounted, two-photon imaging, multiphoton, behavior, cortex, rat, brain imaging, *in vivo* imaging

## Abstract

Rats are used in neuroscience research because of their physiological similarities with humans and accessibility as model organisms, trainability, and behavioral repertoire. In particular, rats perform a wide range of sophisticated social, cognitive, motor, and learning behaviors within the contexts of both naturalistic and laboratory environments. Further progress in neuroscience can be facilitated by using advanced imaging methods to measure the complex neural and physiological processes during behavior in rats. However, compared with the mouse, the rat nervous system offers a set of challenges, such as larger brain size, decreased neuron density, and difficulty with head restraint. Here, we review recent advances in *in vivo* imaging techniques in rats with a special focus on open-source solutions for calcium imaging. Finally, we provide suggestions for both users and developers of *in vivo* imaging systems for rats.

## 1 Introduction

Advances in genetically encoded sensors provide increased sensitivity, cell type specificity, and the ability to record a variety of signals from intracellular calcium^1^ and membrane voltage,^2^ to neurotransmitter release such as dopamine.^3^^,^^4^ New microscopes have been developed to image across larger areas, with greater resolution, increased depth, and enhanced portability.^5^^–^^7^ These methods are being increasingly paired with sophisticated analytical techniques, which have opened new avenues within theoretical neuroscience.^8^^–^^10^

**Fig. 1:**
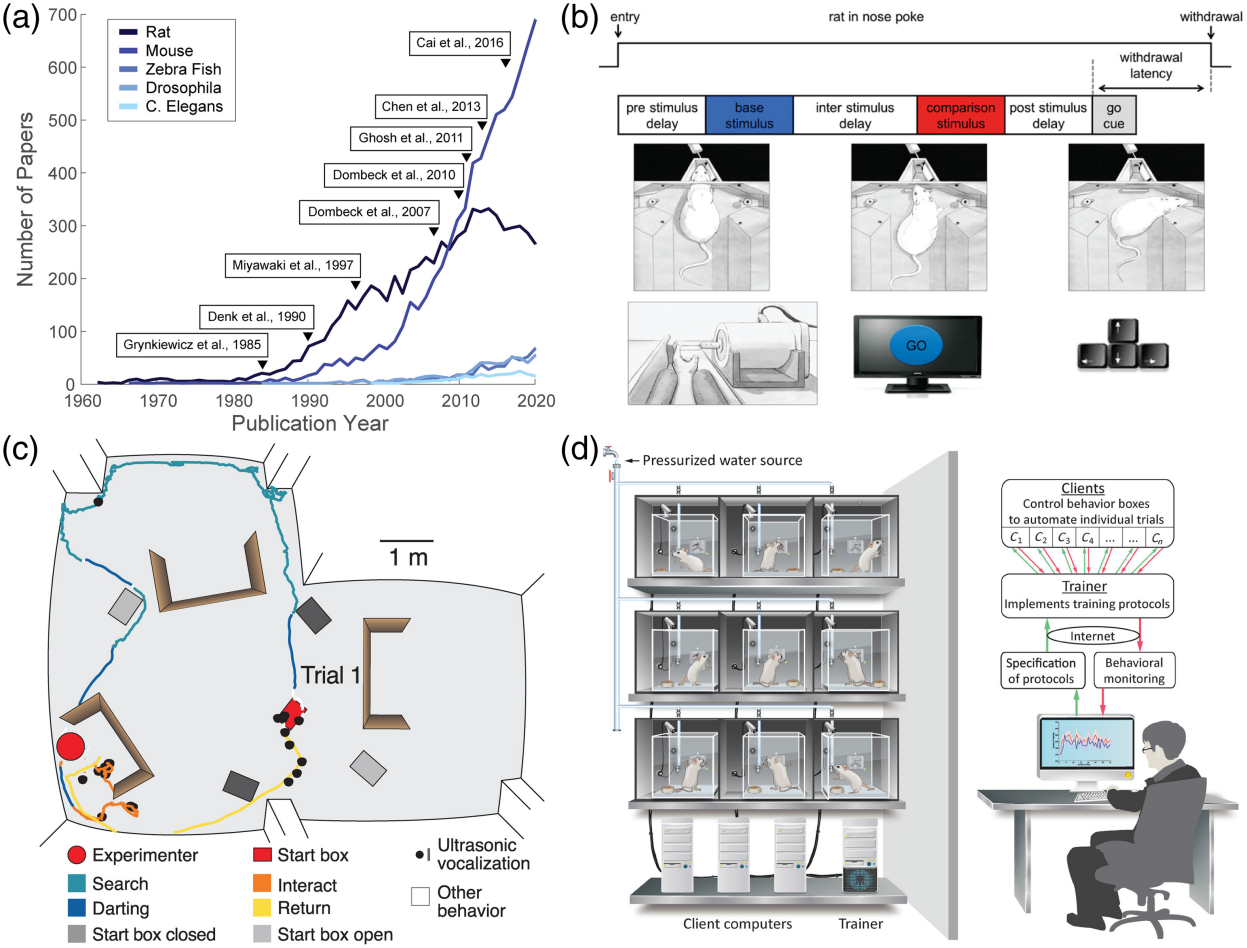
Tasks and behavioral control systems used in rats. (a) Number of papers on PubMed by year with the search term “calcium imaging” and either “rat,” “mouse,” “zebrafish,” “drosophila,” or “*Caenorhabditis elegans*” from 1964 to 2019. Key calcium imaging papers are denoted by a triangle and the citation: development of fura-2, a fluorescent dye to detect calcium,^12^ the first 2P microscope,^13^ development of an early genetically encoded calcium sensor,^14^ a treadmill system for *in vivo* imaging,^15^ the first VR system used with 2P imaging in mice,^16^ wearable epifluorescent microscope,^17^ development of GCaMP6,^18^ and development of the open-source miniscope.^19^ (b) Schematic of a tactile comparison task to measure parametric working memory (top), with rats (middle) and humans (bottom) performing the task.^20^ (c) Trajectory of a hide-and-seek task trial in rats, where the rat emerges from the start box and searches for the human experimenter.^21^ (d) A fully automated, live-in facility for rat behavioral training.^22^

The development of *in vivo* cellular resolution imaging technologies, and calcium imaging in particular, has been one of the modern success stories in systems neuroscience.^11^ Over the past 60 years, these tools have been applied to a variety of model organisms [Fig. 1(a)]. However, in the last 15 years, the mouse has emerged as a leading model for *in vivo* cellular resolution imaging. This is likely due to the confluence of genetic tools, such as transgenic mouse lines (e.g., Ref. 23), and methods that enable imaging during behavior, such as head-fixed virtual reality (VR^16^) and head mounted microscopes.^17^

While success of *in vivo* imaging technologies in mice has inspired the field, progress in other organisms, including rats, continues. Rats have historically been an important model for biomedical and neuroscience research (Refs. 24 and 25; see Table 1). Today they remain a leading model for studying neural dynamics during complex learned behaviors, such as navigation, decision making, and addiction. The behavioral advantages of this organism have motivated continued innovation in applying calcium imaging tools. Recent successes reflect this: new imaging technologies for rats include multiphoton microscopy using voluntary head restraint,^34^^,^^35^ open-source widefield microscopes for large field of view (FOV) recording,^36^^,^^37^ head mounted three photon (3P) microscopes,^38^ and transgenic rats expressing calcium indicators.^34^^,^^37^

**Table 1:** Pioneering discoveries in systems neuroscience using the rat model.

Discovery	Reference(s)
Adult neurogenesis	Altman and Das^26^
Place cells	O’Keefe and Dostrovsky^27^
Head direction cells	Taube et al.^28^
First BOLD measurement with fMRI	Ogawa et al.^29^
Odorant receptor gene	Buck and Axel^30^
Neural replay	Wilson and McNaughton^31^
*In vivo* 2P imaging	Denk et al.^32^
Grid cells	Hafting et al.^33^

Given the significance of the rat animal model in neuroscience and neuroimaging specifically, continued development of *in vivo* imaging tools in this species is warranted. This review will focus on specific opportunities and challenges posed by neuroimaging in the rat model, describe the technical solutions under development, and provide an outlook for technologies that may facilitate future imaging experiments.

## 2 Opportunities in Rat Imaging

The rat model has advantages that motivate its continued use for studying the link between cellular dynamics and behavior. In this section, we provide an overview of these advantages and the experimental opportunities of the rat model system.

### 2.1 Behavioral Repertoire

Rats distinguish themselves as model organisms because of their complex behavioral repertoire, adaptability, and the variety of tools to study both learned and natural behaviors. Rats can be trained on a wide range of tasks designed to characterize goal-directed behaviors and decision-making.^39^^–^^43^ For example, rats can readily learn to perform parametric working memory tasks inspired by primate tasks^20^^,^^44^ [Fig. 1(b)] and can learn the representation of action-outcome associations in a multi-step planning tasks.^45^^,^^46^ Rats can learn behavioral paradigms originally developed for humans, facilitating comparative studies and translational research in neuropsychiatry.^47^^,^^48^ Rats are also social creatures,^49^ demonstrating pro-social behaviors in controlled laboratory environments,^50^^,^^51^ including empathy,^52^ cross species play^21^ [Fig. 1(c)], and collaborative group search.^53^

The wide range of behavioral features in rats contribute to their usefulness as a model organism for basic and translational neuroscience research. Unfortunately, direct, quantitative comparisons of behaviors between rats and other model organisms, in particular mice, is rarely performed, and this limitation is particularly acute in complex decision-making tasks, which are presently of great interest.^54^ Ethological behaviors are somewhat conserved; mice and rats have similar aggression, grooming, feeding, and reproductive behaviors.^55^ While the overall behavioral patterns are consistent between species, there are slight nuances to many of these innate behaviors (e.g., rats exhibit more complex grooming phases than mice). A quantitative comparison between rat and mouse behavior across a range of tasks would facilitate an unbiased assessment of the pros and cons of each species. In some cases, such as addiction, these side-by-side comparisons have been performed. For example, there is some indication that rats are a better model for studying alcohol relapse behaviors than mice.^56^

Numerous open-source tools and pipelines have been developed for behavioral training and measurement in rats. These include VR navigation systems^57^^–^^59^ automated operant systems^22^^,^^39^ [Fig. 1(d)], touchscreen training,^60^ and voluntary head restraint.^61^^–^^63^ Together, the availability of experimental and computational tools for behavioral research in rats provides frameworks for collecting and analyzing high-throughput data in a variety of laboratory settings, which can easily be paired with multimodal imaging approaches.^64^

### 2.2 Body Size

Adult rats weigh hundreds of grams (250 to 350 g for a 10 week old male Long Evans)^65^ and have significant capacity for implantable and wearable devices. Rats can carry head mounted devices weighing 35 g while still displaying natural behaviors, such as rearing and rapid head orienting.^37^ This capacity reduces constraints on development allowing for microscopes with larger FOVs^36^^,^^37^ and or more complex optical components.^66^ Beyond the rat’s physical strength, the larger size and rectilinear shape of the skull provides ample “real estate” for device attachment.

Beyond the technological advantages that rats provide because of their physiology, rats can also act as a bridge to larger model organisms for neuroscientific research. As we describe below, the brains of larger animals pose challenges to imaging, which will require new imaging capabilities. Rats, with their relatively wide range of available transgenic lines and genetic tools, may provide a valuable test case for developing and expanding technology for other animals, such as ferrets, macaques, and marmosets.

## 3 Challenges in Rat Imaging

### 3.1 Head Restraint

Head restraint is widely used in neuroscience to stabilize the brain position relative to the imaging apparatus. Head restraint in rats can be accomplished through an acclimation process in which the duration of restraint is gradually increased.^67^ However, compared with mice, this approach is unreliable and more limited in rats—they show increased stress and diminished behavioral flexibility during head restraint.^68^ Consequently, forced head restraint is not frequently used in conjunction with complex cognitive task learning in rats. This has motivated the development of head-mounted microscopes and voluntary head-fixation (see Sec. 4).

### 3.2 Decreased Neuronal Density

While being 8 to 10 times the body mass of mice, rats have three times the number of neurons; much of this increase in neurons is in the cerebellum, and the fraction of cortical neurons remains constant even as total brain size increases.^69^^–^^71^ Mice have on average 78,672 neurons and 68,640 nonneuronal cells per milligram of cortical tissue, whereas rats have 41,092 neurons and 60,430 nonneuronal cells per milligram.^69^ In terms of density, rats have half the number of neurons per milligram of cerebral cortex compared with mice.^70^^,^^72^ Lower neuron densities will result in fewer imaged neurons when assuming the same FOV and signal-to-noise ratio (SNR). This challenge is not unique to rats—it is a challenge shared by many larger-brained animals, including several primate species.^69^^,^^70^^,^^73^

### 3.3 Increased Cortical Thickness

The rat neocortex is thicker than the mouse neocortex; for example, the motor cortex of rats has an average thickness of 1.6 mm while in mice motor cortex has an average thickness of 1.0 mm.^71^ Since the scattering length of the rat cortex is similar to that of the mouse,^32^^,^^74^^–^^78^ the excitation light penetrates to a comparable depth in both animals. Overall, this results in reduced optical access into deeper layers in the rat brain. In most cases, cell somas in layer 2/3 of rat neocortex, which ranges from 200 to 500  μm,^79^ can lie below the range of some head-mounted one-photon imaging systems^17^ and makes imaging of infragranular layers difficult. To surpass these limitations, researchers can implant microprisms, relay gradient index (GRIN) lenses, or use 3P microscopy, all three of which we discuss in more detail in the following section (see Sec. 4).

### 3.4 Vascular Size and Branches

Rat brains have an increased number of capillary branches per unit volume and larger radii of vessels compared to mouse brains.^80^^,^^81^ This can lead to changes in the optical properties of tissue, such as increased absorption of light at different wavelengths due to hemoglobin.^37^^,^^82^^–^^84^ In addition, these differences in vasculature can contribute to difficulties in surgery (such as increased bleeding) when compared to mice.

### 3.5 Transgenesis

Tools for the production of transgenic rats are well developed and several lines of genetically modified rats that express calcium sensors have been produced (see Sec. 4). However, the costs, speed of generation, and number of off the shelf transgenic lines in mice greatly exceeds the rat model at present. The availability of transgenic lines is an important feature that should be considered when selecting a model organism for calcium imaging studies.

## 4 Tools for Rat Imaging

Below, we highlight the recent applications of imaging tools and labeling techniques in rats.

### 4.1 Transgenic Lines

Several useful transgenic lines for neuroscience and specifically *in vivo* imaging are available from several sources, including the Rat Resource and Research Center (RRRC) and the Rat Genome Database.^85^ Available lines include Cre driver lines for cell type specific expression (e.g. Refs. 86 to 88) and genetic models for human neuropsychiatric disorders, such as models of autism.^89^ The Rat Genome Database provides a valuable list of resources for the development of transgenic rats.^90^

**Fig. 2:**
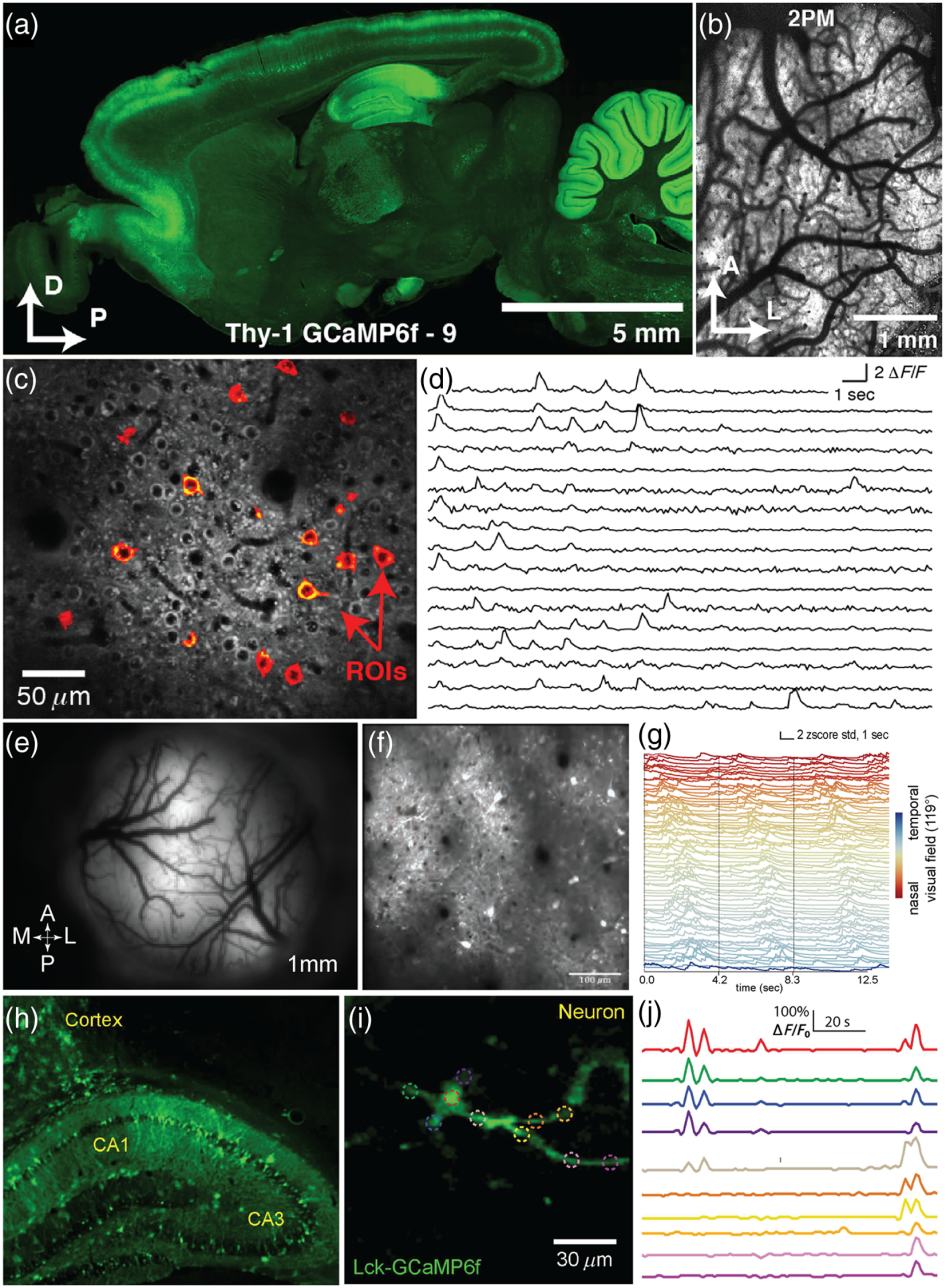
Labeling systems for rats. (a) Sagittal section of a Thy1 GCaMP6f-9 rat (from Ref. 37). (b), (c) 2P imaging of layer 2/3 of the cerebral cortex of a transgenic rat expressing GCAMP6f, where red pixels identify ROIs.^37^ (d) Calcium traces from the 17 ROIs in panel C at 30 Hz.^37^ (e) Epifluorescence image of a cranial window in a rat following serial viral injections with AAV9-GCaMP7f.^67^ (f) 2P imaging of a 500  μm×500  μm FOV from rat cortex injected with GCaMP7f.^67^ (g) Z-scored traces from the rat visual cortex for three cycles of presentation of a moving bar sweeping in the nasal-to-temporal direction at 0.24 Hz. Traces are colored and sorted by the corresponding cell’s phase at the stimulation frequency.^67^ (h) Confocal imaging of a coronal section of the rat hippocampus expressing Lck-GCaMP6f following *in utero* electroporation.^91^ (i) Mean calcium activity projection of a neuron expressing Lck-Gcamp6f following *in utero* electroporation and using 2P microscopy.^91^ (j) Calcium traces from the same cortical neuron, with colors corresponding to the dashed ROIs in panel (i).^91^

Transgenic lines have also been developed that express genetically encoded calcium sensors for *in vivo* imaging^34^^,^^37^ [Figs. 2(a)–2(c)]. These lines, created by Janelia Research Campus on the Long-Evans background, express the genetic calcium indicator GCaMP6f throughout large regions of the CNS, with different transgenic lines having clusters of expression in different areas.

Sensor expression in at least two transgenic rat strains, Thy-1-GCaMP6f-7 and Thy-1-GCaMP6f line 8, is sufficient for cellular resolution imaging through either one or two-photon (2P) microscopes.^34^^,^^37^^,^^92^ However, the use of newer GCaMP variants delivered by adeno associated viral vectors (AAV) injection appears to provide improved SNR and action potential (AP) detection. For example, Chornyy et al.^93^ found that single AP detection was detected in 10.6% (48/450) of GCaMP6f-labeled neurons labeled in Thy-1-GCaMP6f animals, whereas it was detected in ∼85% (412/485) of jGCaMP7s-positive cells labeled with AAVs. These results indicate that new GCaMP variants and/or viral labeling may improve signal detection.

### 4.2 Viral Vectors

At the time of writing, the majority of published studies involving imaging of genetically encoded sensors in rats express sensors using direct injection of AAVs^35^^,^^36^^,^^63^^,^^94^^–^^98^ [Figs. 2(d) and 2(e)]. AAVs are favored due to the high-levels of expression that are difficult to obtain in transgenics^99^ and the availability of new genetically encoded sensors, which are being developed more rapidly than new transgenic lines. Direct injection of high-titer viral vectors into the rat CNS is widely used to achieve local expression of genetically encoded sensors. To achieve more widespread expression, several alternative approaches have been explored. One method is using serial injections, which has been demonstrated across the rat cortex. In this approach, a series of injections are performed at regular increments, tiling a larger volume. This approach aims to achieve a more uniform labeling over a larger volume than could be achieved by a single injection [Figs. 2(d) and 2(e)].^67^^,^^88^^,^^100^ Several groups have also reported widespread CNS infection in adults following systemic administration through intravenous,^101^^–^^103^ intraventricular, and intrathecal injection.^104^^,^^105^ These techniques reduce the potential for damage to neural tissue following direct injections. The efficiency of these techniques is enhanced by the development of enhanced AAV capsids (such as PHP.eB), which yield improved gene transfer in rat CNS.^104^^,^^106^ While these approaches are intriguing, they have not been widely used in combination with *in vivo* functional imaging approaches in rats.

### 4.3 *In Utero* Gene Delivery

Another method for gene delivery used in rats is via *in utero* electroporation, a method for transfecting neural tissue with plasmid DNA via injection into embryonic brains [Figs. 2(f) and 2(g)]. *In utero* electroporation enables widespread expression in neurons throughout the CNS.^91^^,^^107^^–^^111^ In addition, *in utero* AAV injections can be used to achieve widespread cortical labeling in rats.^112^

A strength of *in utero* gene delivery is that it can be implemented during different stages of development to yield spatially specific expression within the neocortex without the need for laminar specific promoters. Moreover, the method can be optimized to produce widespread infection from a single injection. That said, gene delivery to the rat embryo requires specialized techniques and equipment, and there is some indication that introduction of foreign genetic material during development can produce an immune response that alters or even damages the brain.^113^

### 4.4 Head-Mounted Microscopes Designed for Mice

Miniaturized head mounted epifluorescence microscopes allow recording of calcium dynamics in freely behaving animals.^17^ This approach bypasses the problem of head restraint and stabilization while achieving cellular resolution imaging.^114^ These microscopes have been widely used in mice, but several groups have applied these miniature microscopes in rats.^92^^,^^94^^–^^98^^,^^115^ However, performance in these scopes is often optimized for mice. For example, early generations of UCLA microscopes have an FOV of $∼1\;{\mathrm{mm}}^{2}$. Next generation miniscopes designed with rats in mind have a larger FOV to account for the decreased cell density in the species (discussed in greater detail below).

The size and strength of the rat can create issues for the physical stability of head-mounted microscopes. Open-source systems, such as headcap covers, have been developed to protect and stabilize the scope.^116^ A headcap system for protecting the microscope also permits a solution for reducing movement-related torque on the microscope from the tethering cable. Once implanted, an anchoring point on the headcap offset from the microscope can be used to fix the tether to the headcap and thus reduce force transferred at the connection point with the microscope.

### 4.5 Microprisms

**Fig. 3:**
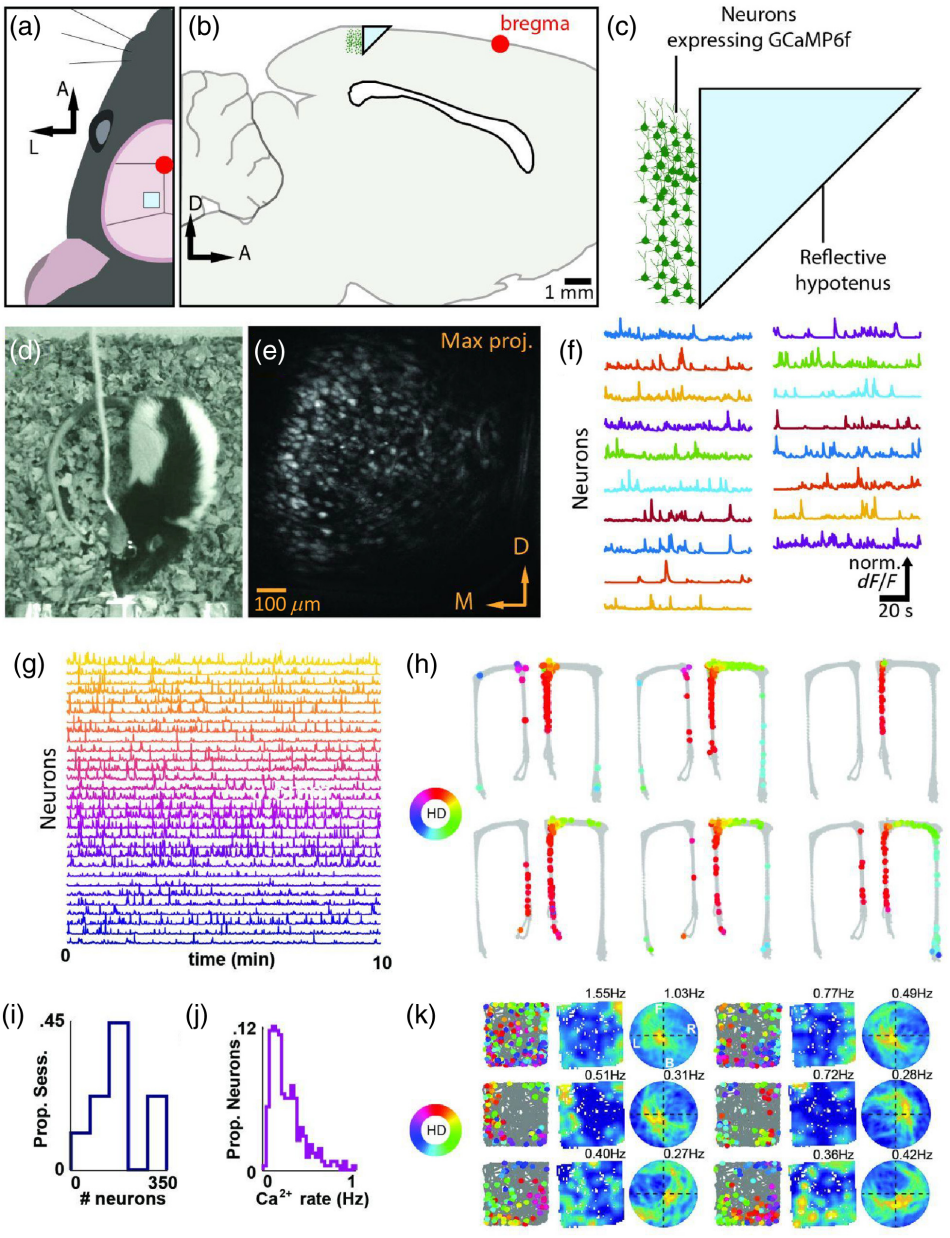
Calcium imaging in transgenic rats through implanted microsprisms. (a) Position of the implanted microprism for imaging in the rat RSC relative to the rat head. (b) Schematic of the implantation location in a sagittal section. (c) Schematic of the prism imaging approach. (d) Image of an implanted rat wearing a head mounted one-photon camera in an operant chamber. (e) Maximum intensity projection from the imaging FOV. (f) Example time traces from selected ROIs from E showing fluorescence transients during an operant-based task. (g) Deconvolved ${\mathrm{Ca}}^{2+}$ traces from 30 simultaneously recorded RSC neurons. (h) Six RSC neurons, recorded using this preparation, exhibit differential activation for different trajectories on a delayed alternation spatial working memory task on a T-maze. Gray lines represent trajectory on track, split into leftward and rightward trials. Colored dots indicate animal position and head direction at the time of a calcium transient. Color indicates head direction according to legend on top right. (i) Number of cells per session. (j) Distribution of mean transient rate from a single recording. (k) Simultaneous recording of six RSC neurons with egocentric boundary vector responsivity. (Left) Trajectory plot with animal path in gray and spike locations indicated in colored circles where color is animal heading orientation in the environment. (Middle) Two-dimensional ratemap of “spiking” activity. (Right) Egocentric boundary ratemap showing position of boundaries at time of calcium transient. F, front; B, behind; R, right; L, left. All plots are maximum normalized (blue = zero activity, yellow = maximal).

As discussed, rat cortex is thicker relative to mouse cortex and this increased depth increases light scattering, decreases SNR, and prevents optical access to deep layers. One way to bypass these issues is to image through microprisms implanted directly into neural tissue as previously reported in mice.^117^^,^^118^ Recently, Alexander et al.^92^ successfully paired microprisms with head-mounted one-photon microscopes to image large populations of neurons in rat neocortex (Fig. 3). In this preparation, a $1\;{\mathrm{mm}}^{2}$ microprism attached to a relay lens was positioned near neurons expressing GCaMP6f to create an FOV perpendicular to the dorsal surface of the brain spanning multiple cortical layers [Figs. 3(a)–3(c)]. A baseplate was attached to the skull above the microprism, which allowed a head-mounted microscope to be mounted [Fig. 3(d)]. Using this preparation, it was possible to simultaneously monitor calcium dynamics of hundreds of neurons with robust SNRs in Thy1-GCaMP6f transgenic rats performing track running or free exploration [Figs. 3(e)–3(k)]. Well known spatial coding properties of the retrosplenial cortex (RSC) were replicated using this method in rats including trajectory-dependent coding [Fig. 3(h)] and coding for environmental boundaries in egocentric coordinates [Fig. 3(k)].

### 4.6 Head-Mounted 1P Microscopes Designed for Rats

**Fig. 4:**
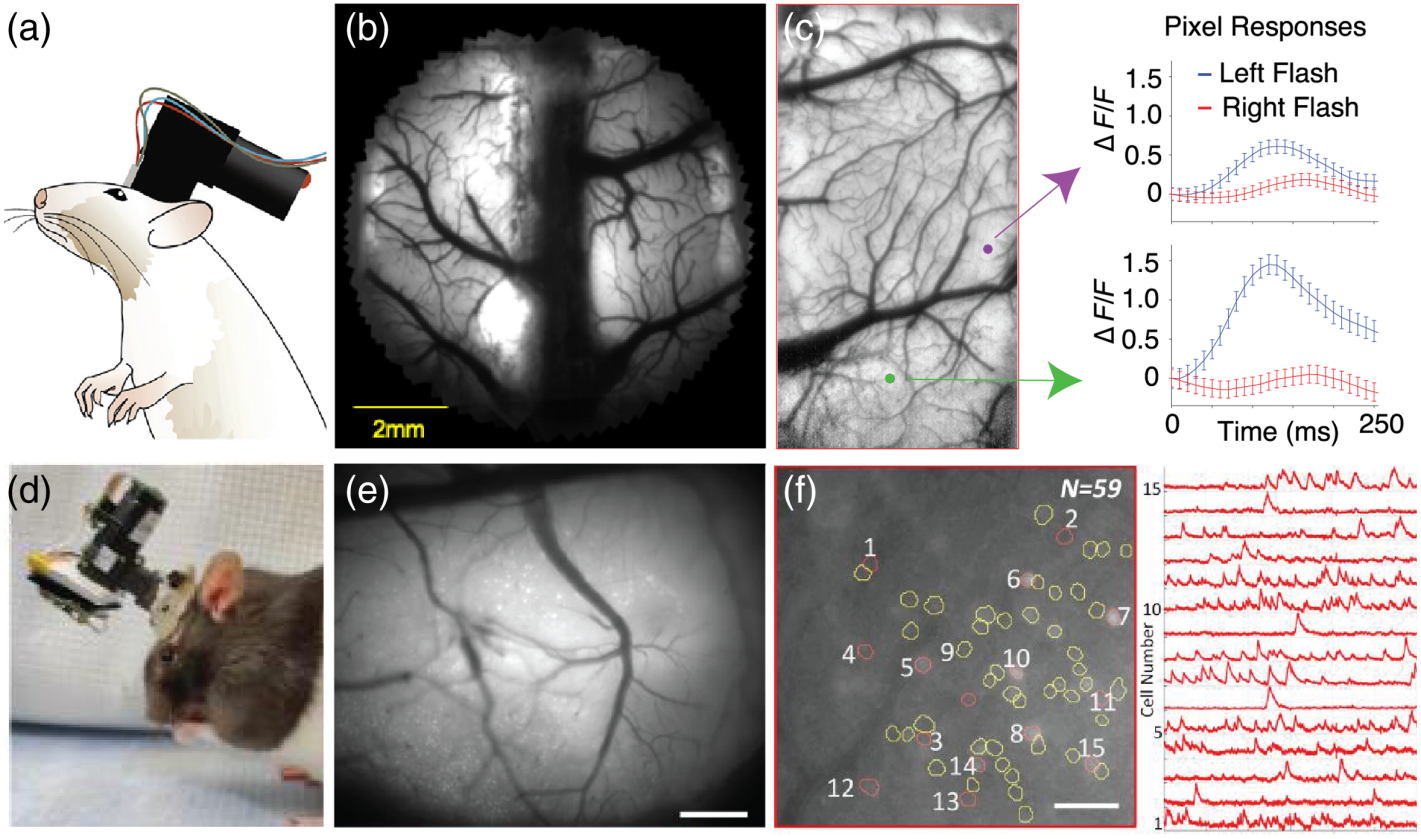
Head mounted widefield microscopes designed for rats. (a) Schematic of a rat wearing cScope, a head-mounted widefield macroscope.^37^ (b) Image of the FOV in a rat implanted with cScope. (c) Left: cScope fluorescence image, with colored dots indicating the location of the pixels that contribute to the responses on the right. Right: Flash response dynamics of the corresponding single pixel ROIs. (d) Picture of a rat wearing MiniLFOV.^36^ (e) Maximum projection of a motion-corrected recording session. Scale bar: 500  μm. (f) Left: Map within panel (e) of 59 cells. Scale bar: 100  μm. Right: Calcium traces from a subset of 15 cells within panel (f) across 6 min.

Head-mounted widefield microscopes with larger FOVs have been developed for rats (Fig. 4). Larger FOVs enable the monitoring of larger populations of neurons and permit the examination of cross-regional dynamics not afforded by a smaller FOV targeting a single brain region. Previously, researchers developed cScope, a head mounted widefield macroscope to access FOVs up to $8\;{\mathrm{mm}}^{2}$ [Figs. 4(a)–4(c)].^37^ cScope uses a hemodynamic illumination collar with green LEDs for reflectance illumination of cortical intrinsic signal and a blue LED for fluorescence imaging. Recordings using cScope have similar performance compared to conventional widefield epifluorescence microscopes, with imaging frame speed up to 30 Hz. However, the authors did not report cellular resolution calcium dynamics or whether this fluorescence signal originates from soma or neuropil.

A recent implementation of the UCLA Miniscope, Miniscope-LFOV, was developed for rats [Figs. 4(d)–4(f)].^36^ This system is a one-photon microscope, which has two electrically adjustable working distance (±100  μm) configurations that allow for cortical imaging via a cranial window and deep brain imaging via a relay GRIN lens. It has a 3.6  mm×2.7  mm FOV, with one FOV in CA1 revealing 1357 cells.^36^ The SNR in this microscope is considerably higher when compared with the performance of previous Miniscope iterations, attributable to newer and more sensitive detection systems in Miniscope-LFOV compared to its Miniscope predecessors. Recently published work details a system for online data pre-processing with Miniscope-LFOV,^119^ enabling researchers to perform motion correction, calcium trace extraction, and recognize neural patterns, which are correlated to behavior.

### 4.7 Head-Mounted Multiphoton Microscopes Made for Rats

**Fig. 5:**
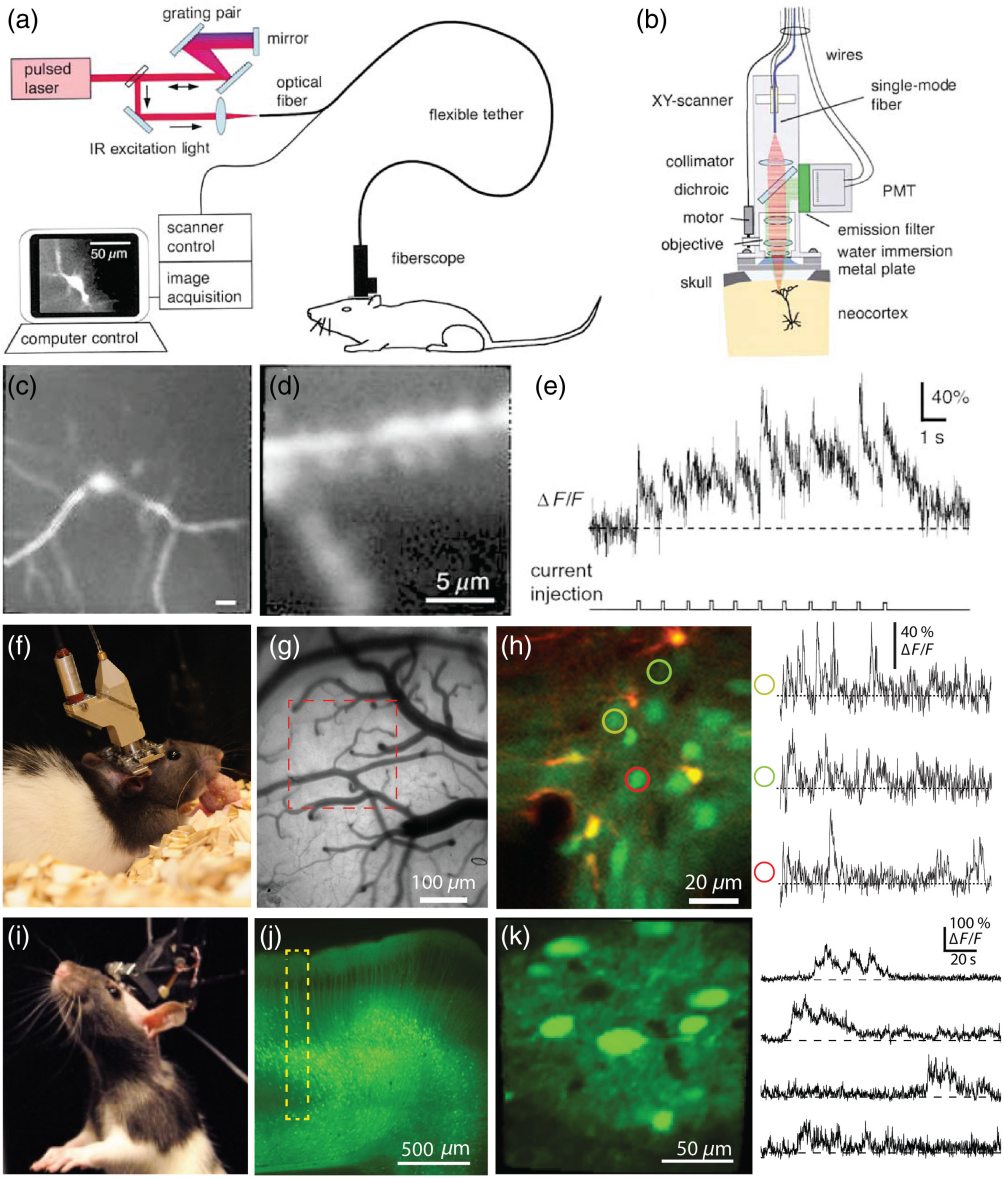
Head-mounted multiphoton microscopes used in rats. (a) Diagram of the light path and setup of the first head-mounted 2P microscope.^120^ (b) Schematic of the internal components in the fiberscope design. (c) Images of somatosensory cortex L2/3 neurons filled with calcium green-1. (d) Zoomed in image of a different dendrite from in somatosensory cortex L2/3. (e) Example calcium green-1 fluorescence trace along a dendritic process following current injection at 1 s intervals, with 10 ms resolution. (f) Picture of a rat wearing a head-mounted 2P microscope.^66^ (g) Camera image of the primary visual area, with the 2P imaging sites identified with the red dashed line. (h) Left: Two color 2P imaging of primary visual cortex using sulforhodamine 101 and OGB1-AM. Right: calcium time courses of the soma of three neurons (colored circles in the left panel) across 30 s. (i) Image of a 120 g rat wearing a head mounted three-photon microscope.^38^ (j) Histological section of GCaMP6s-labeled neurons in posterior parietal rat cortex, with the yellow dotted box showing the attainable imaging depth (1120  μm). (k) Left: Labeled neurons at 1120  μm depth below the cortical surface. Right: Example spontaneous calcium kinetics from FOV on left.

The carrying capacity of rats has facilitated the development of advanced head-mounted microscopes, such as multiphoton microscopes. The first head-mounted 2P microscope was developed for rats in the early 2000s, by Helmchen et al. [Figs. 5(a)–5(e)].^120^ This microscope was 25 g in weight and 7.5 cm in height. Scanning was achieved by a fiber tip that resonated to form a Lissajous pattern. More recent iterations allow for increased performance, including raster scanning, and provide optical access to deeper areas with cellular resolution imaging in behaving rats [Figs. 5(f)–5(h)].^66^ Today head mounted 3P microscopes for rats have cellular resolution as deep as 1.1 mm with a 150  μm square FOV [Figs. 5(i)–5(k)]^38^ and more recently adapted to mice.^121^

Like their tabletop counterparts, head-mounted multiphoton microscopes have several key features that facilitate calcium imaging *in vivo* in larger brained mammals, such as rats. The longer excitation wavelengths allow for less scattering in tissue and greater power delivery at depth. The non-linear properties of excitation provide optical sectioning and a reduction in out-of-focus excitation from fluorescence contamination from sources above and below the imaging plane.^13^^,^^122^ Multiphoton imaging can improve the ability to resolve cellular structures like axonal projections and dendrites in scattering tissue and can reduce contamination from the neuropil *in vivo*.^123^ However, head-mounted multiphoton microscopes are still outperformed by table top microscopes, including both commercial and custom systems, due to fewer space and weight constraints in the tabletop environment. Therefore, in order to combine the power to table top scopes with automated behavioral training systems, voluntary head restraint tools have been developed.

### 4.8 Voluntary Head-Restraint

**Fig. 6:**
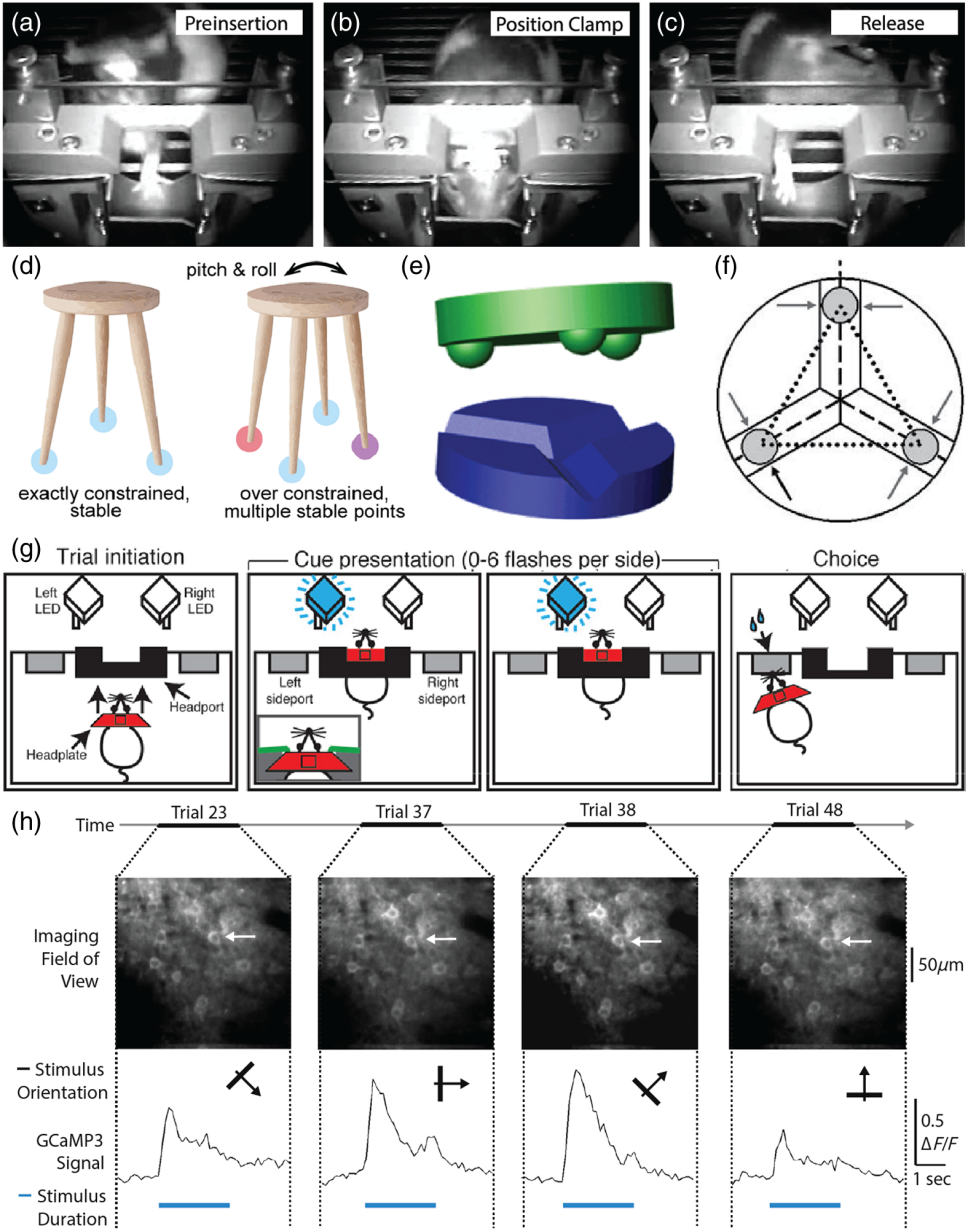
Principles of voluntary head restraint. (a)–(c) A rat voluntarily head restraining across three stages: pre insertion, positioning of the head clamp and fixation, and release.^63^ (d) The principles of kinematic coupling. Objects can be exactly constrained with stable points equal to the degrees of freedom the object has, or over-constrained such that there are multiple stable points possible. Kinematic coupling enables high degrees of repeatability and accuracy by exactly constraining objects.^124^ (e) Toy model of a vee groove kinematic clamp.^124^ (f) Diagram of the degrees of freedom constrained in a vee groove kinematic clamp.^125^ (g) Behavioral paradigm schematic where rats are trained to voluntarily head restrain during an evidence accumulation task.^42^ (h) 2P imaging of GCaMP3-labeled cortical neurons across several voluntary head-restraint trials. Top panels show V1 without motion correction. Bottom panel shows fluorescence transients from the selected neuron (indicated by the white arrow). On each trial, a visual stimulus was presented with differently oriented drifting gratings as denoted by the black arrow, with the blue line underneath indicating time of visual stimulus presentation.

Voluntary head-restraint is a system in which trained rodents submit to periods of mechanical head restraint for reward (Fig. 6). Initially developed for rats for repeatable presentation of visual stimuli,^61^^,^^62^ demonstrations that computer controlled training systems for precise positioning and stability catalyzed renewed interest in voluntary head restraint.^63^^,^^126^ Work in rats inspired researchers to develop automated behavioral systems using voluntary head-fixation in mice.^127^^–^^130^ These head fixation systems have been designed for mechanical stability and repositioning within several microns and to be used together with widefield imaging or optogenetics.

Researchers have adapted voluntary restraint systems for cellular resolution population calcium imaging in behaving rats.^34^^,^^35^^,^^63^ These systems used kinematic clamps to achieve high repositioning accuracy and produce the mechanical stability required by cellular resolution imaging [Figs. 6(d)–6(f)]. Kinematic clamps^131^^,^^132^ are commonly used in optical and mechanical systems to achieve precise and repeatable alignment. To this end, recent work demonstrates that head fixation devices with micron-scale and submicron-scale repositioning accuracy for cellular resolution imaging are feasible.^124^ These systems improved upon previously published Kelvin-style kinematic coupling systems^63^ by utilizing a three vee-groove system, also known as a Maxwell system, which is simpler to manufacture and enables greater long-term performance.^133^ The design principles described have been scaled up to evaluate voluntary head restraint in larger animals.^134^

**Fig. 7:**
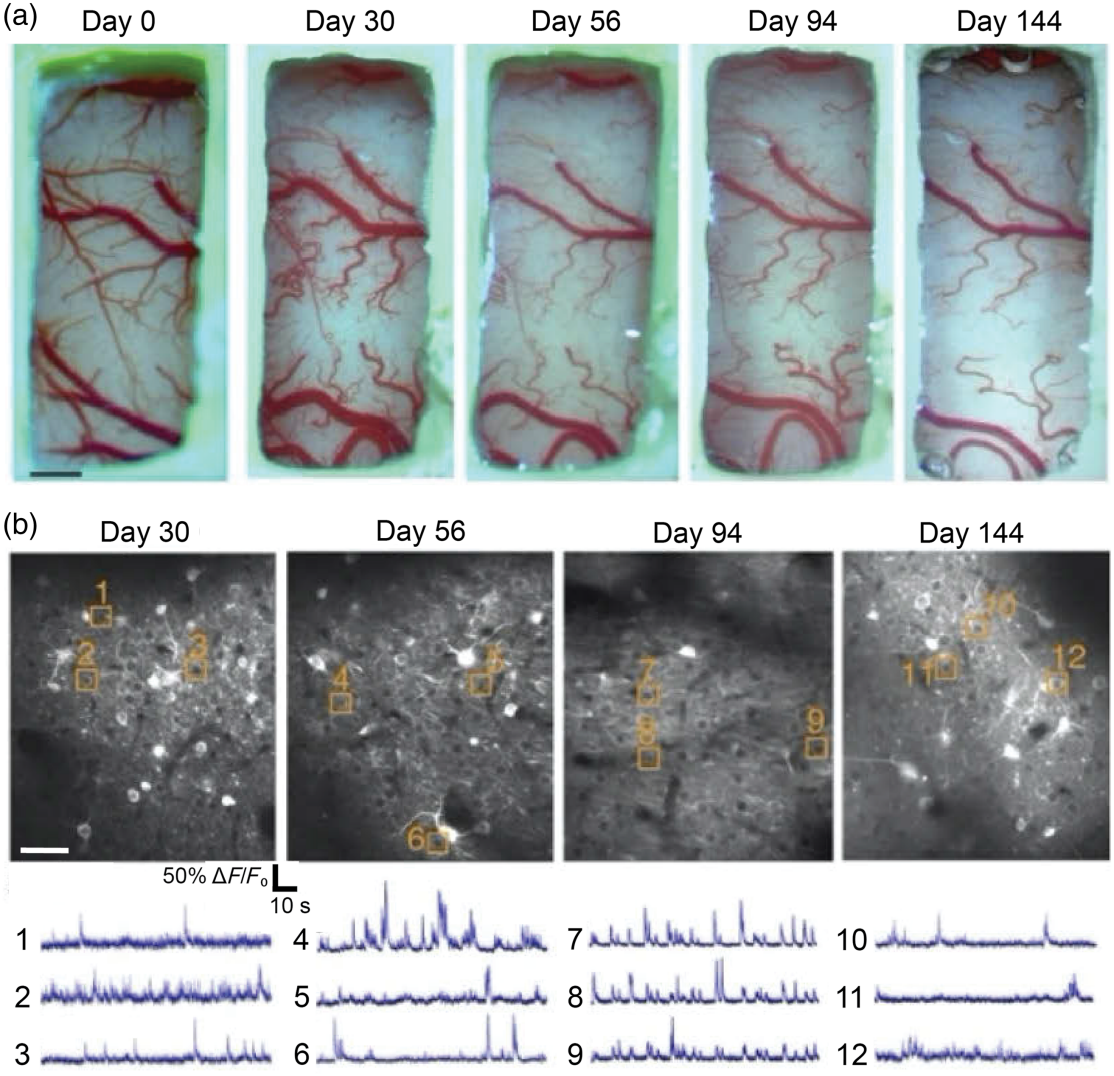
Longitudinal 2P imaging in rats. (a) Brightfield images of the same cranial window in a rat, beginning from day of implantation (day 0). (b) 2P images of jGCaMP7s-expressing neurons with ROIs and the corresponding spontaneous activity traces from the somatosensory cortex of the same rat in (a). Note that the window quality remained high over 144 days, as reflected in the clarity of the window in the brightfield images.^93^

Recent work demonstrates the potential of combining voluntary head-restraint with transgenic rats to record neuron population dynamics over long timescales.^34^ In this study, a new line of transgenic rats were reported to express GCaMP6f at high levels in hippocampal neurons. These rats were implanted with a newly developed magnetic-based kinematic coupling system and trained in voluntary restraint. Upon becoming proficient, animals performed hundreds of daily fixations over multiple months. 2P imaging through an implanted optical cannula over hippocampal CA1 provided the ability to track a large population of hippocampal neurons for well over a year. Other long term imaging preps (over 140 days) can also be achieved with viral labeling^93^ (Fig. 7) and with fluorescent dextran (98 days).^135^ We point out that each of these three groups removed the dura, and future studies will be required to evaluate the impact of different surgical preparations on longitudinal imaging in rats. These studies demonstrate the potential for longitudinal imaging in rats, which could be valuable for experiments on aging, plasticity, and representational drift.

## 5 Outlook

Below, we highlight future directions that may improve cellular resolution imaging in the rat model and may help experimentalists determine if the rat model is appropriate for their research program.

### 5.1 Next Generation Optical Design

Next generation imaging systems for rats may be improved by increasing the imaging depth, increasing the FOV of imaging systems, and enhancing the SNR to account for the physiological limitations discussed above. The use of 3P imaging can help compensate for the increase in cortical thickness and enable the recording of neuronal activity down to layer 5,^38^ whereas the use of a large FOV instrument may compensate for the reduction in cell density. The combination of the two, which has been recently described,^136^^,^^137^ could enable activity recording from large neuronal populations in the rat.

Computational approaches have been used to reduce out-of-focus fluorescence neuropil contamination^123^ and suppress measurement noise in calcium imaging data.^138^^,^^139^ Aside from improving the quality of the data, reduction of noise and out-of-focus light can potentially enable deeper imaging in the rat brain. Computational methods may also aid with the development of new imaging systems. Software designed to simulate the optical, anatomical, and physiological properties of the mouse brain^140^ may allow for rapid development of next generation imaging systems and provide a standardized ground truth for evaluating their performance. Extending this simulation tool to rats would be a valuable next step and should be feasible given the extensive physiological data available.^69^^,^^70^^,^^80^^,^^81^

### 5.2 Imaging in Cellular Compartments

Several new molecular genetic approaches could be considered in order to improve imaging performance in rats. For example, neuropil contamination could be reduced by expression of soma restricted calcium sensors.^141^^,^^142^ In addition, simultaneous imaging of multiple cell types could be achieved by restricting sensors to readily differentiable cellular compartments, such as axons and soma. Finally, imaging of apical dendrites could allow access to deep cortical neurons, an approach used to support population imaging in macaques.^143^

### 5.3 Multi-Device Imaging

The larger size of the rat loosens spatial constraints with neuroimaging methods. One way would be to incorporate multiple head-mounted microscopes targeting different regions, akin to *in vivo* electrophysiology. This approach has been applied in mice by targeting two distant regions of interest (ROIs) by developing a smaller one-photon microscope configuration.^144^^,^^145^ Multiple off-the-shelf head-mounted microscopes could be situated on the rat skull using angled, longer relay lenses; this would enable proper clearance for the microscope and lens attachment.

A similar method could be utilized to pair *in vivo* neuroimaging, *in vivo* electrophysiology, or perturbation methods in freely behaving rats. As a consequence of a greater working area, ferrules or cannulae could be positioned in areas outside of the imaging window, counter to current methods that record calcium dynamics and provide optogenetic stimulation within the same FOV. Calcium activity of large neural ensembles or neuromodulatory dynamics in one region could be compared with respect to electrophysiological activity—including oscillatory dynamics—in another area.^115^ Neuroimaging signals in the same FOV could be compared before and after optogenetic or pharmacological manipulations to another structure.

## 6 Conclusion

Extending neuroscience tools to a diverse set of species will allow researchers to study how the brains of different species solve similar biological problems.^146^ This is synergistic with new priorities for cross-species comparative work, in which similar behavioral methods and recording tools are applied across multiple species.^147^^–^^149^ Expanding technologies to organisms beyond the species that the technology was originally developed poses a significant challenge. It is our hope that rats can serve both as a valuable model for systems neuroscience and act as a bridge to new framework for applying *in vivo* imaging tools more broadly across a diverse set of species.

## Data Availability

Data sharing is not applicable to this article, as no new data were created or analyzed.
